# In Vitro and In Vivo Anti-inflammatory Activity of Bovine Milkfat Globule (MFGM)-derived Complex Lipid Fractions

**DOI:** 10.3390/nu12072089

**Published:** 2020-07-15

**Authors:** Kate P. Palmano, Alastair K. H. MacGibbon, Caroline A. Gunn, Linda M. Schollum

**Affiliations:** 1 Retired from Fonterra Research & Development Centre, Palmerston North 4442, New Zealand; kate.palmano@xtra.co.nz; 2 Fonterra Research & Development Centre, Palmerston North 4442, New Zealand; Caroline.Gunn@fonterra.com (C.A.G.); Linda.schollum@fonterra.com (L.M.S.)

**Keywords:** MFGM, phospholipids, gangliosides, dairy, inflammation, polar lipids, anti-inflammatory, IL-1β, nitric oxide, superoxide anion, cyclo-oxygenase-2, neutrophil elastase

## Abstract

Numerous health related properties have been reported for bovine milk fat globule membrane (MFGM) and its components. Here we present novel data on the in vitro and in vivo anti-inflammatory activity of various MFGM preparations which confirm and extend the concept of MFGM as a dietary anti-inflammatory agent. Cell-based assays were used to test the ability of MFGM preparations to modulate levels of the inflammatory mediators IL-1β, nitric oxide, superoxide anion, cyclo-oxygenase-2, and neutrophil elastase. In rat models of arthritis, using MFGM fractions as dietary interventions, the phospholipid-enriched MFGM isolates were effective in reducing adjuvant-induced paw swelling while there was a tendency for the ganglioside-enriched isolate to reduce carrageenan-induced rat paw oedema. These results indicate that the anti-inflammatory activity of MFGM, rather than residing in a single component, is contributed to by an array of components acting in concert against various inflammatory targets. This confirms the potential of MFGM as a nutritional intervention for the mitigation of chronic and acute inflammatory conditions.

## 1. Introduction

Fat droplets in milk are enclosed in a thin triple-layer membrane, called the milk fat globule membrane (MFGM), which stabilises the droplets as a colloidal dispersion throughout the liquid and prevents them from coalescing [1,2]. The MFGM is a complex association of both protein and lipid components and in particular, is a rich source of polar lipids such as phospholipids, sphingolipids and gangliosides [3]. The membrane proteins are distinct from milk serum proteins and comprise only 1–2% of the total milk protein. There are several dairy streams containing higher levels of MFGM than fresh milk and these can be used to produce various extracts with specific enrichments in either the protein or polar lipid components [4,5,6]. 

In recent years, the MFGM has garnered much interest as a specialty ingredient, due to the numerous health benefits associated with the bioactivities of both its protein and lipid constituents (for reviews see [1,7]). In animal and human trials, positive health outcomes have been reported following dietary intake of MFGM and in particular its polar lipid-enriched fractions, including enhanced neural and cognitive development [8,9], improved brain function and neuroplasticity [10], enhanced postnatal neuromuscular development [11], anti-infective and anti-febrile activity in infants [12,13] and postprandial cholesterol modulation [14]. Additionally, MFGM isolates or fractions have been shown to have anti-inflammatory properties in vitro [15,16,17], in animal models [14,18,19], and human clinical trials [14,20,21], including protection against intestinal inflammation in neonatal rat pups [22] and low birth weight mouse pups [23]. Furthermore, safety and tolerability of MFGM for use in infant formula has been confirmed [7]. 

It is likely that some of these bioactivities can be associated with specific components, or classes of components, which are present in the MFGM [24]. For example, sphingolipids and their various metabolites have been shown to have anti-cancer, anti-infective, anti-cholesterolaemic and gut maturation properties, in particular, by modulating the inflammatory responses associated with the various pathologies (for reviews see [1,3,25,26,27,28,29]). Dietary phospholipids have also been demonstrated to have an array of bioactivities including protection against cardiovascular disease, chemopreventive and chemotherapeutic activities, enhancement of memory and cognition, and anti-inflammatory activity in diseases such as arthritis and inflammatory ulcerative colitis (reviewed in [1,8,25,26,30]). Further, lack of expression of bacteria- derived phospholipids has been demonstrated to increase intestinal inflammation [31].

Clearly MFGM has the potential to confer a spectrum of health benefits, based on the activities of individual components or components acting in concert. However, its anti-inflammatory activity is of interest as inflammation is an underlying factor in many adverse health outcomes and diseases [32], and most chronic inflammatory diseases as well as allergic diseases are strongly influenced by nutrition [33]. There is some indication from the literature that differentially enriched MFGM fractions may deliver different anti-inflammatory outcomes. Park et al., [18] showed that a ganglioside enriched milkfat (MFGM) supplement inhibited release of pro-inflammatory signals in the intestinal mucosa and blood in a rat model of acute gut inflammation. Using in vitro models of acute gout, Dalbeth et al. [15] demonstrated that the MFGM-derived ganglioside-enriched dairy fraction G600, but not MFGM-derived phospholipid enriched fraction PC500, decreased pro-inflammatory cytokine release from THP-1 cell lines. In addition, the G600 concentrate inhibited the influx of inflammatory cells in a murine urate peritonitis model [15]. In a recent dietary intervention trial, however, a beta serum powder (BSP) that was lactose depleted [34], was shown to have no effect on post-prandial pro-inflammatory markers in obese and overweight adults [35]. This suggests that dose and composition could both be important. 

In this paper, we present novel in vitro and in vivo data which provide further insights into the anti-inflammatory activity of various MFGM preparations. In an attempt to elucidate the most effective anti-inflammatory components of the MFGM, we tested a range of isolates derived from the beta serum starting material, each with varying concentrations of bioactive components, against a panel of in vitro assays of inflammatory biomarkers, with certain fractions being further selected for screening in well-established animal models of acute and chronic inflammatory arthritis. 

## 2. Materials and Methods 

### 2.1. Analytical Assays

The phospholipid concentration was determined by 31P nuclear magnetic resonance spectroscopy (NMR) as described by MacKenzie et al. [36]. The ganglioside concentration was determined by liquid chromatography high-resolution electrostatic ion-trap mass spectrometric (HPLC-MS) analysis as described by Fong et al. [37].

### 2.2. MFGM Fractions

MFGM complex lipid products BSP, BPC70, PC500, PC600, PC700, G500 and G600 were provided by Fonterra Co-operative Group Ltd., Auckland, New Zealand. Table 1 gives the gross composition of each of these products and indicates the various enrichments in phospholipid and ganglioside content. The MFGM isolates that were tested were concentrates derived from a beta serum powder (BSP) from anhydrous milkfat production. The manufacturing flow has been described by Gallier et al. [4] and Fontecha et al. [6]. The P) series (PC500, PC600, PC700) are phospholipid concentrates (PC) with varying degrees of residual neutral lipid (milkfat). The G series (G500, G600) are a more polar ganglioside concentrated fraction which includes a greater concentration of the more polar phospholipids. BPC70 is a MFGM protein dominant fraction produced by supercritical extraction using carbon dioxide and dimethylether [34].

### 2.3. Animal Procedures

All animal procedures and in vivo experiments were approved by the Wellington School of Medicine Animal Ethics Committee, NZ, according to national guidelines and regulations on animal welfare at the time of the individual studies. Animals were housed in conventional facilities with temperature control (20 ± 2 °C), a 12h light/dark cycle, and ad libitum access to food and water. Rats were euthanised with intraperitoneal ketamine/xylamine immediately prior to blood sampling at the end of each trial, or if necessary, during a trial for humane reasons. 

Rat Monocytes were isolated for nitric oxide (NO) and interleukin -1β(IL-1β) assays as described in (Current Protocol in Immunology, John Wiley & Sons, Inc. Hoboken, NJ, USA.) (2001) [38].

Rat Neutrophils were isolated for neutrophil elastase (NE) and superoxide anion (SO) production assays as described [39]. Whole blood taken by cardiac puncture from healthy male Dark Agouti (DA) rats was collected into heparinised tubes and layered over Polymorphprep™ (density 1.113 g/mL; Alere ASA, Oslo, Norway). After centrifugation at 500× *g* for 30 min at 20 °C the lower of the two visible leukocyte bands, containing polymorphonuclear cells was harvested, washed twice and resuspended to a concentration of 10^7^ cells/mL in Hanks Balanced Salt Solution (HBSS). Cells were maintained on ice and used within 2–4 h of collection. Purity of the cells as assessed by cytocentrifugation and staining was >95%.

### 2.4. In Vitro Assays

MFGM test samples were initially solubilised in 20% ethanol in HBSS (Hanks Balanced Salt Solution, Gibco Laboratories, Grand Is, NY, USA) to form stock solutions of 10 mg/mL. Samples were further diluted as necessary in 20% ethanol in HBSS. Unless otherwise stated the samples were tested at 1:100. 1:200 and 1:400 dilutions and measured in triplicate.

In vitro assays were principally cell and whole blood-based, and targeted the inflammatory mediators Interleukin-1β (IL-1β), nitric oxide (NO), superoxide anion (SO), cyclo-oxygenase-2 (COX-2), and neutrophil elastase (NE), all of which have key roles in the inflammatory responses underlying numerous chronic health conditions and pathologies [8,40,41,42,43,44,45,46]. Data on cyclo-oxygenase-1 (COX-1) activity are also included to indicate COX selectivity. Unlike inducible COX-2, COX-1 is not involved in acute inflammatory responses but is a constitutive enzyme involved in gastric mucosal protection [47].

#### 2.4.1. Measurement of Neutrophil Elastase Activity

Assay of NE in activated rat neutrophils was based on the method of Yoshimura et al. [48]. PMA (phorbol 12-myristate 13-acetate, Sigma Aldrich, St Louis, MO, USA) was used to activate the cells, Alpha-1 proteinase inhibitor (Sigma-Aldrich, St. Louis, MO, USA) was used as a positive control and N-methoxysuccinyl-ala-ala-pro-val p-nitroaniline (N-A) (Sigma-Aldrich, St. Louis, MO, USA) was used as the chromogenic enzyme substrate in the assay. 

#### 2.4.2. Measurement of Superoxide Anion Production

Measurement of SO production by PMA-activated neutrophils was based on the colorimetric assay of Tan and Berridge ([49] in which a chromogenic substrate, the tetrazolium salt WST-1 (PreMix WST-1 Cell Proliferation Assay System, Takara Bio Inc., Shiga, Japan), is oxidised by SO produced during the respiratory burst. Aspirin (Sigma-Aldrich, St. Louis, MO, USA) dissolved in HBSS was used as a positive control. 

#### 2.4.3. Measurement of Nitric Oxide Production

This assay measured the release of NO following LPS-stimulated monocyte to macrophage conversion (Current Protocols in Immunology, Chapter 14: Oxidative metabolism of murine macrophages Unit 14 15. John Wiley & Sons, Inc., 2001) [50] and was based on the methods described in Yoshimura et al. [48] The NO concentration was measured by the colorimetric Griess reagent procedure using a kit according to the manufacturer’s instructions (Cat No. 23479, Sigma, St Louis, MO, USA). LPS (Sigma-Aldrich, St. Louis, MO, USA) was used as the positive control for NO, and L-NMMA (NG-Methyl-L-Arginine, Sigma-Aldrich, St. Louis, MO, USA) was used as an inhibitor for NO. 

#### 2.4.4. Measurement of Interleukin -1β Release

This assay measured the release of the cytokine IL-1β following LPS-stimulated monocyte to macrophage conversion, as described in Current Protocols in Immunology, Chapter 14: Oxidative metabolism of murine macrophages, Unit 14 15., John Wiley & Sons, Inc., 2001 [50]. 

Indomethacin (Sigma-Aldrich, St. Louis, MO, USA) was used as the positive control. IL-1β was measured using an ELISA kit (R & D systems, Cat. No. RLB00) following the manufacturer’s instructions.

#### 2.4.5. Measurement of COX-1 and COX-2 Activity

COX-1 and COX-2 activities were determined using either a whole rat blood assay [51,52]) or using human cell line U937 monocytes ([53]. The human monocyte U937 cell line was purchased from American Type Culture Collection (ATCC, Rockville, MD, USA). Production of thromboxane B2 (TXB2,) or prostaglandin E2 (PGE2) following stimulation with LPS, were used as measures for COX-1 and COX-2, respectively. Indomethacin was used as a positive control. Both markers were determined using ELISA kits according to the manufacturer’s instructions: TXB2 ELISA kit (R & D Systems, Minneapolis, MI, USA; Cat. No. DE0700), PGE2 ELISA kit (Cat. No. DE0100, R & D Systems, Minneapolis, MI, USA). 

Final assay concentrations of MFGM test samples were 5–500 μg/mL. Controls and test compounds were assayed in duplicate.

#### 2.4.6. Measurement of Human Neutrophil Elastase Activity

The effect of MFGM fractions on the in vitro activity of HNE was measured using the release of *p*-nitroalinine from the chromogenic substrate N-A. This assay was based on the methods described in [48]. p-Nitroaniline release was measured spectrophotometrically at 405 nm using a Bio-Rad ELISA reader.

### 2.5. In Vivo Experiments

#### 2.5.1. Adjuvant-Induced Rat Model of Rheumatoid Arthritis

MFGM preparations were assessed for their abilities to modulate joint inflammation using the adjuvant-induced model of joint swelling in rats [54,55]. Control and treatment groups each comprised 8 female DA rats, aged 21 to 24 weeks (Trial A), or 6 female DA rats aged 16–19 weeks (Trial B), at the commencement of the study. Rats were fed either normal AIN-93 diet (control groups) or a diet supplemented with MFGM preparations at 38.3mg/g of diet (treatment groups) for two weeks prior to challenge [55]. On the same day as adjuvant injection, a positive control group was established by daily administration of an anti-arthritic drug via oral gavage (0.12 mg meloxicam/kg body weight (Metacam®, Boehringer-Ingelheim, St. Joseph, MO, USA)), until the completion of the trial 17 days after CFA administration.

Rat weights were recorded throughout the trial and food consumption was recorded every three days. From the 10th day after CFA administration, the size of each hind foot and ankle was measured daily with a plethysmometer. The volume measured was used to determine the foot volume change relative to the control at 17 days. Clinical scoring of the inflammation was also carried out, using the American Rheumatism Association guidelines [56,57] and a modification of the clinical severity scoring systems used by Larsson et al. [58] and Kawahito et al. [59]. Scores were assigned as follows: (A) for the three joints of the lateral four fingers or toes, 0 = normal, 1 = swelling; (B) for midfoot, mid-forepaw, ankle and wrist joints, 0 = normal, 1 = mild swelling, 2 = moderate swelling, 3 = severe swelling, 4 = non-weight bearing. The individual rat arthritic score was obtained by summing the scores recorded for each limb to give the foot score. The foot score measures the clinical severity of the inflammation, thus a score of zero is the normal condition. Maximum score per animal was 80. 

#### 2.5.2. Carrageenan-Induced Rat Model of Acute Inflammation 

MFGM preparations were assessed for their abilities to modulate carrageenan-induced acute inflammation in the hind foot-pads of rats using 100 µL of 2.5% (*w*/*v*) carrageenan (Type IV lambda, Sigma Aldrich St Louis, MO, US) [60,61]. Each study group comprised 4 male Lewis (300–325 g) and 4 female Lewis rats (240–260 g). Rats were fed either normal AIN-93 diet (control groups) or diet supplemented with MFGM preparations at 38.3 mg/g of diet (treatment groups) for 15 days prior to the induction of acute inflammation. Food consumption and weight were recorded every three days. A positive control group was established by administering meloxicam by oral gavage on each of the two days prior to carrageenan injection. Paw swelling as determined by the volume displacement of the hind feet of all rats using a plethysmometer, was measured prior to, and 4 h after carrageenan injection. The percentage increase in volume of each foot was calculated. 

#### 2.5.3. Statistical Analyses 

Statistical analyses were aimed at combining data from different experiments to obtain representative estimates of anti-inflammatory activity of MFGM preparations at different dose levels. In short, sample results were firstly expressed as percentage of their respective control (% Control), results from different trials were then pooled using weighted means, and lastly the significance of the pooled results was evaluated using the student *t*-test.

Results from the original experiments were recorded in a centralised repository as summary data, namely group mean, number of replicates, and standard deviation (SD) or standard error of the mean (SEM). Most of the group means were recorded in the original units but in some cases, data were only available as percentage of the control value (% Control). The detailed procedure, as follows, was applied to both in vitro and in vivo data: (1) mean sample results for each product (and the inhibitor control if included) were expressed as % Control using the mean of the control from the respective experiment. The SD of % Control was calculated from the approximate variance of the sample-to-control ratio using Taylor expansions [62]. (2) Where the same preparation was tested in multiple experiments, % Control results were pooled by calculating weighted means and SDs [63]. (3) The two-sided t-value for the difference between pooled mean and 100% was calculated (using the total number of replicates (n) to calculate the SEM of %Control) and its *p*-value found from the t distribution for n-1 degrees of freedom [63]. For preparations where only % Control data (mean, SD, and *p*-value) was originally recorded, results are reported accordingly herein, with or without pooling. Statistical significance for pooled results was evaluated as under (3).

All calculations were performed using Microsoft Excel 2016 and significance is declared if *p* < 0.05.

## 3. Results

### 3.1. In Vitro Assays

The effect of MFGM fractions on the production or activity of the various inflammatory mediators are presented below. Values are expressed as % control or as % stimulated control (LPS+, or PMA+) in those assays where cells were activated to produce an inflammatory response. Indomethacin, alpha-1-proteinase inhibitor, L-NMMA and aspirin were all effective inhibitors in their respective target assays, with aspirin inhibiting superoxide production in a dose-dependent manner. Since indomethacin gave similar levels of inhibition in both the cell and whole blood-based COX assays, inhibitor data from both methods of assay were pooled for COX-1 and COX-2, respectively. It must be noted that cell based in vitro assays are inherently variable, batch to batch and day to day, due to changes in the live cell concentrations and activity. To mitigate this the results have been expressed as % of the control activity to make comparison easier. Even so, variation was seen in the % of control response of the positive controls (especially where experiments were pooled). Thus while trends are observed, absolute results were more variable (as seen in the error bars).

#### 3.1.1. Neutrophil Elastase (NE)

Results for NE activity following activation of rat neutrophils were collated from two datasets which are distinguished from each other as Series 1 (PI-1) and Series 2 (PI-2). 

All the MFGM products tested appeared to inhibit the activity of NE. The first dataset shows that BSP, G500 and G600 all gave similar levels of inhibition, while the response with PC600 appeared to be more marked.

**Figure 1:**
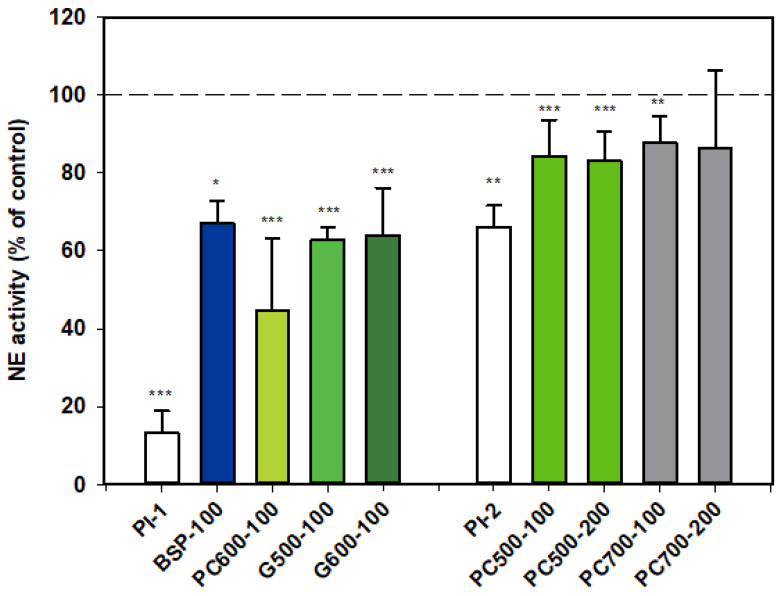
Neutrophil elastase activity relative to the control, two datasets. Values are means and SD (represented by error bars). Different coloured bars refer to different ingredients as indicated on the bottom axis (as described in Methods section and Table 1), with the last set of digits on each label indicating the ingredient dose (µg/mL). The positive control α-1-proteinase inhibitor (PI, 100 µg/mL) is shown prior to the samples in each series (PI-1 and PI-2 referring to series 1 and 2). Values for Series 1 represent the means from 1 experiment with triplicate measurements, or pooled means from 2 independent experiments, each with triplicate measurements. Values for Series 2 represent the pooled means from 2 or 3 independent experiments, each with triplicate measurements. The first series represents data where only % Control (mean, SD, and *p*-value) was originally recorded. Significance relative to control, * *p* < 0.05; ** *p* ≤ 0.01; *** *p* ≤ 0.001.

PC500 and PC700 were also mildly inhibitory, but as alpha-1 proteinase inhibitor appeared to be much less effective in this assay set, levels of inhibition for these two fractions cannot be directly compared with those of the other MFGM fractions (Figure 1).

#### 3.1.2. Superoxide Anion (SO)

**Figure 2:**
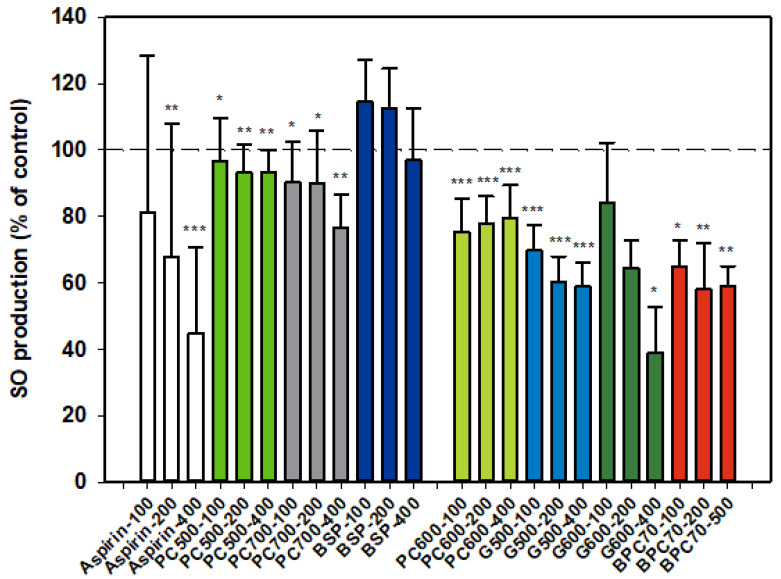
Superoxide anion production relative to the control. Two dose-response data sets. Values are means and SD (represented by error bars). Different bar colours refer to different ingredients as indicated on the bottom axis (as described in Methods section and Table 1), with the last set of digits on each label indicating the ingredient dose (µg/mL). The first data set includes aspirin as a comparison. The second set represents data where only % Control (mean, SD, and *p*-value) was originally recorded. Values for the first data set represent either the means from one experiment with triplicate measurement, or the pooled means from 3–4 independent experiments with triplicate measurement. Values for the second dataset represent either the means from one experiment with triplicate measurement, or the pooled means from 2 independent experiments with triplicate measurement. Significance relative to control, * *p* < 0.05; ** *p* ≤ 0.01; *** *p* ≤ 0.001.

Results for SO production by activated neutrophils were collated from two dose-response datasets, and as for NE, one set is distinguished from the other (Figure 2). Aspirin inhibitor values were consistent between datasets and the overall dose response shown in the first dataset (Figure 2) is representative of both sets. The MFGM total isolate BSP had no significant effect on SO production by neutrophils while the higher protein BPC70 extract gave significant, although similar, levels of inhibition at all three doses tested. The patterns of response of SO production to phospholipid enriched extracts were very similar, with generally mild inhibition across the dose range but no well-defined dose response. PC600 was slightly more inhibitory than either PC500 or PC700. The two ganglioside enriched fractions were also effective at inhibiting SO production. A weak dose response was observed with G500 with inhibition being significant at all doses. While a stronger dose response was observed with G600, the results were significant only at the highest dose (400 µg/mL). The level of inhibition corresponded to that produced by aspirin at the same dose (Figure 2).

#### 3.1.3. Nitric Oxide (NO)

**Figure 3:**
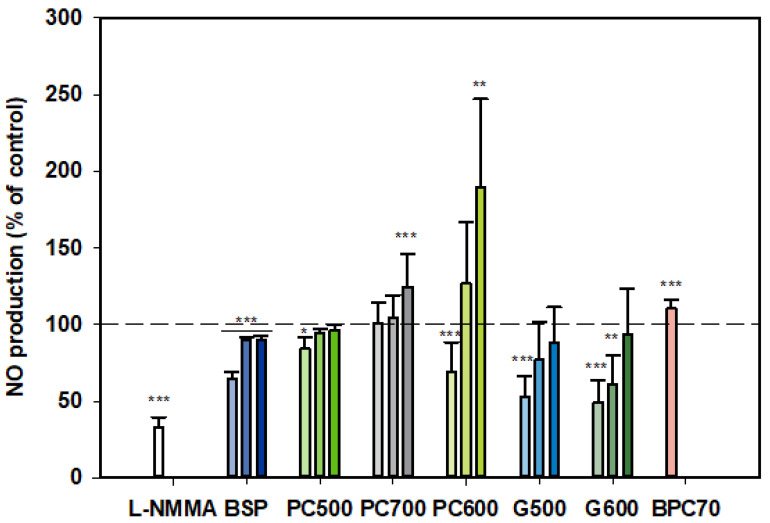
Nitric oxide production dose responses for samples relative to the control. Values are means and SD (represented by error bars). Different bar colours refer to different ingredients as indicated on the bottom axis (as described in Methods section and Table 1). Doses shown as filled bars (light, medium and dark) for 100, 200, 400 µg/mL, respectively. The BPC70 dose is 100 µg/mL. Positive control was L-NMMA (1 mM final concentration). Values represent either the means from one experiment with triplicate measurement, or the pooled means from 2–3 independent experiments with triplicate measurement. Data for L-NMMA represents the pooled mean from 3 independent experiments. Significance relative to control, * *p* < 0.05; ** *p* ≤ 0.01; *** *p* ≤ 0.001.

At all three doses tested, BSP inhibited NO production by LPS-stimulated monocytes, although the strongest inhibition was observed at the lowest dose (100 µg/mL) (Figure 3). On the other hand, PC700 had no effect at the lower doses while being somewhat stimulatory at the highest dose of 400 µg/mL. BPC70 also appeared to have a slight stimulatory effect, albeit only a single dose was tested. PC500 was mildly inhibitory at 100 µg/mL while PC600 was inhibitory at 100 µg/mL but significantly stimulatory at 400 µg/mL. The ganglioside fractions G500 and G600 both gave similar patterns of response, with the lower doses (100 and 200 µg/mL) being inhibitory (although not significant for G500 at 200 µg/mL), with no significant effect at the highest dose (400 µg/mL). Response at the lowest doses (100 µg/mL) approximated to the positive control, L-NMMA (52.8% ± 13.75 for G500, 49.2% ± 14.29 for G600 vs. 33.2% ± 6.28 for L-NMMA). Notably, this tendency toward an inverse dose response was observed with all fractions where more than one dose was tested (Figure 3).

#### 3.1.4. Interleukin-1ß (IL-1ß)

**Figure 4:**
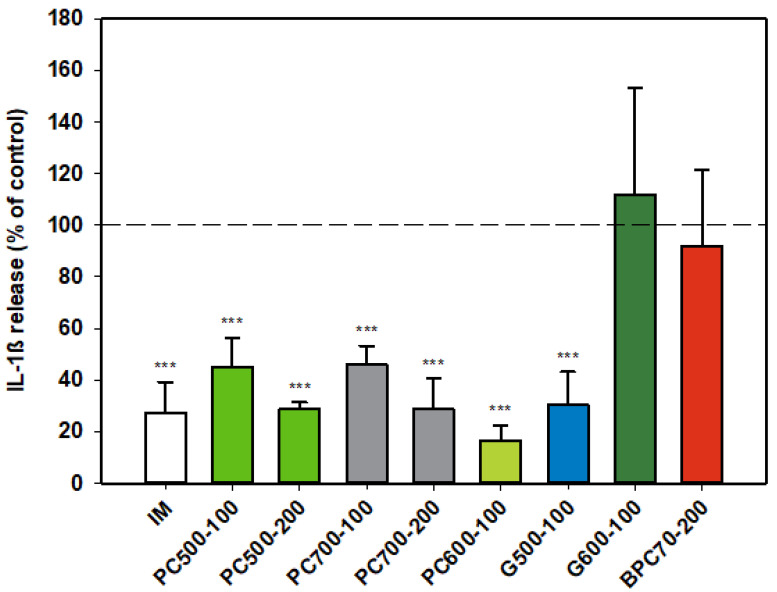
Interleukin-1ß responses relative to the control. Values are means and SD (represented by error bars). Different bar colours represent different ingredients as indicated on the bottom axis (as described in Methods section and Table 1), with the last set of digits on each label indicating the ingredient dose (µg/mL). The positive control is indomethacin (IM, 0.1 mM final concentration). Values represent either the means from one experiment with triplicate measurement, or the pooled means from 2 independent experiments with triplicate measurement. Data for indomethacin represents the mean from one experiment with triplicate measurement. Significance relative to control, *** *p* ≤ 0.001.

The protein enriched fraction BPC70 had no effect on production of IL-1β (Figure 4) from activated rat monocytes, while the phospholipid fractions PC500 and PC700 significantly inhibited the production of the cytokine in an apparently dose-dependent manner. The level of inhibition at the higher dose of 200 µg/mL was similar to that of indomethacin. PC600, the fraction with the highest phospholipid content, was potently inhibitory of IL-1β, while of the two ganglioside concentrates, G500 and G600, only G500 was inhibitory, producing a response similar to that of indomethacin (Figure 4). 

#### 3.1.5. COX-1/COX-2 

**Figure 5:**
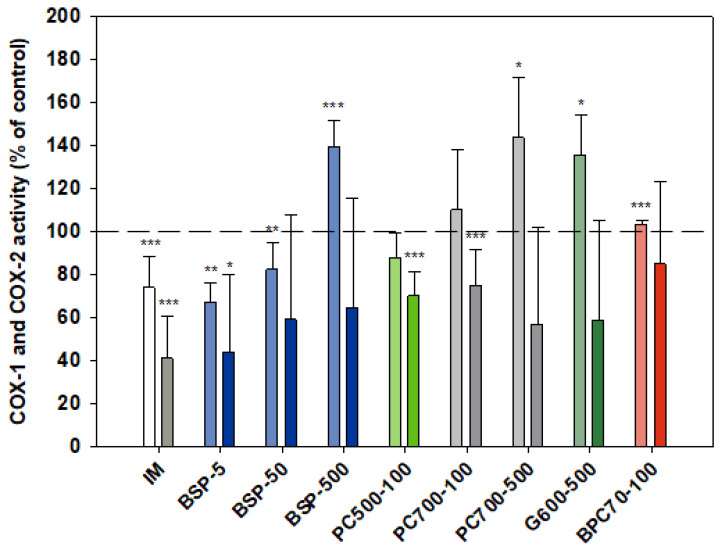
Cox-1 (lighter bars) and Cox 2 (darker bars) responses presented relative to control. Values are means and SD (represented by error bars). Cox-2 response is in the presence of LPS. Different bar colours refer to different ingredients as indicated on the bottom axis (as described in Methods section and Table 1), with the last set of digits on each label indicating the ingredient dose (µg/mL). The positive control is indomethacin (IM, 1 mM final concentration). Values represent either the means from one experiment with triplicate measurement, or the pooled means from 2–4 independent experiments with triplicate measurement. Data for indomethacin represents the mean from 3 independent experiments with triplicate measurement. Significance relative to control, * *p* < 0.05; ** *p* ≤ 0.01; *** *p* ≤ 0.001.

Although indomethacin is known to be a non-selective COX inhibitor with a higher activity against the COX-1 isoform [51,52] in the assays reported here (Figure 5) indomethacin was significantly more inhibitory of COX-2 (than COX-1), *p* = 0.002. As expected, activation of monocytes by LPS (in both cell and whole blood assays) had no effect on COX-1 activity while stimulating COX-2 activity 2.5-fold (not shown). The MFGM isolate BSP was inhibitory of COX-2 at all doses tested over the range 5–500 µg/mL, with a slight inverse dose responsiveness. A stronger inverse dose-response was observed with COX-1, the MFGM isolate inhibiting the enzyme at the lower doses but significantly elevating activity at the highest dose (500 µg/mL). At the lower doses (5, 50 µg/mL) the effect of BSP on both COX isoforms was similar to that of indomethacin. BPC70, the membrane protein-enriched MFGM fraction had no meaningful effect on either of the COX enzymes. The phospholipid-enriched fractions PC500 and PC700, at a dose of 100 µg/mL, were both inhibitory of COX-2 while having no effect on COX-1. PC700 at the higher dose of 500 µg/mL inhibited COX-2, but also significantly increased COX-1 activity. The effect of G600, the ganglioside-enriched MFGM extract, on both COX isoforms was very similar to that of PC700 at the same dose (Figure 5).

#### 3.1.6. Human Neutrophil Elastase (HNE)

**Figure 6:**
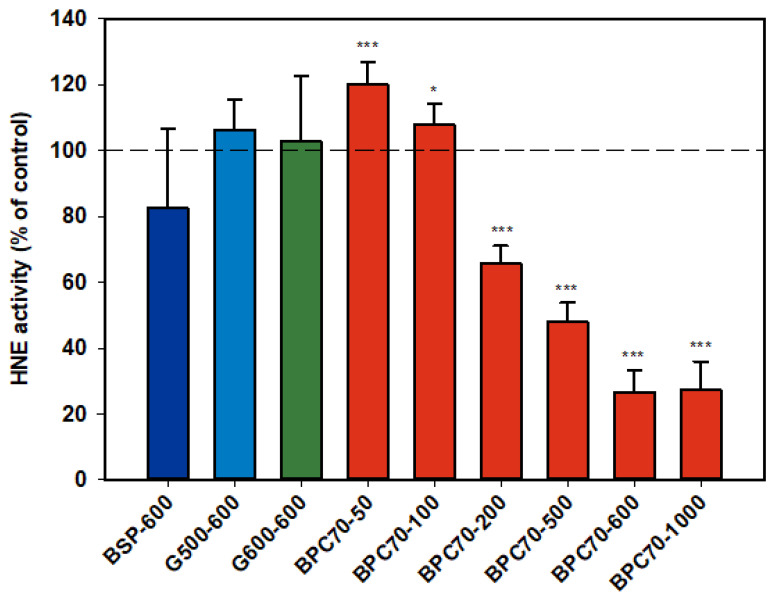
Human neutrophil elastase responses relative to the control. Values are means and SD (represented by error bars). Different bar colours refer to different ingredients as indicated on the bottom axis (as described in Methods section and Table 1), with the last set of digits on each label indicating the ingredient dose (µg/mL). Values represent either the means from one experiment with triplicate measurement, or the pooled means from 2 independent experiments with triplicate measurement. Significance relative to control, * *p* < 0.05; *** *p* ≤ 0.001.

Neither of the ganglioside fractions G500 and G600 had any effect on the activity of the isolated HNE enzyme (Figure 6). The MFGM parent extract, BSP, was not significantly inhibitory, but the protein-enriched BPC70 product demonstrated dose response inhibition between 200–1000 µg/mL, with enzyme activity being inhibited up to 70% at the highest doses. At lower doses (50 and 100 µg/mL), there was some stimulation of the enzyme, but the overall trend was a decrease in enzyme activity with increasing dose (Figure 6).

### 3.2. In Vivo Experiments

#### 3.2.1. Adjuvant-Induced Arthritis

**Figure 7:**
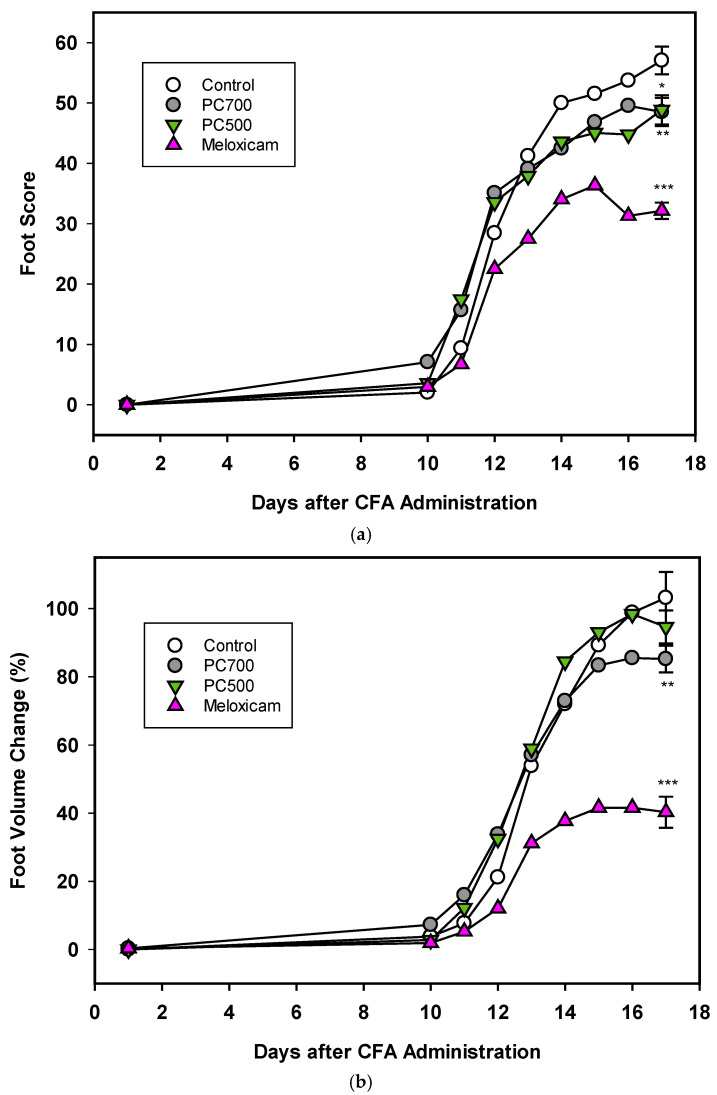
(**a**) The effects of PC700 and PC500 (as described in Methods section and Table 1) in the diet of rats on joint inflammation after the administration of complete Freund’s’ adjuvant (CFA) were compared with a control and the anti-arthritic drug meloxicam, based on the American Rheumatism Association (ARA) Scoring System (foot score). Values are means, n = 8 for all groups except control (*n* = 5). Error bars are SEM and probabilities * *p* <0.05, ** *p* <0.01, *** *p* <0.001. (**b**) The effects of PC700 and PC500 in the diet of rats on joint inflammation after the administration of complete Freund’s adjuvant (CFA) were compared with a control and the anti-arthritic drug meloxicam, based on the change on foot volume (relative to the control at Day 17). Values are means, *n* = 8 for all groups except control (*n* = 5). Error bars are SEM and probabilities * *p* < 0.05, ** *p* <0.01, *** *p* <0.001.

Three of the eight rats in the control group (Trial A) were lost to the trial. In accordance with reported disease trajectories for this animal model [56,64,65,66], clinical symptoms of arthritis were observed 10 days following adjuvant administration and increased rapidly for the next 4 days after which a plateau was reached at around 17 days, as seen in foot scores and foot volumes (Figure 7a,b Trial A), at which time the animals were euthanised. In the group administered meloxicam, inflammatory score and foot volume changes were ameliorated from Day 12 onwards. This was also the case for foot scores for the groups supplemented with MFGM fractions PC500 and PC700 (Figure 7), where a decline in the rate of swelling was observed at Day 12. As also expected with this model, rats lost weight following adjuvant administration with the greatest weight loss occurring between Days 10 and 17, corresponding to onset of clinical manifestations (not shown). Overall mean weight loss was 16–18% for all groups except the meloxicam controls, where mean weight loss was 14.5%. There was no significant difference in food intake between groups over the period of the trial (ANOVA, Tukey HSD post-hoc analysis) and appetite was not affected by weight loss.

The degree of inflammation of the joints at Day 17, the clinical end-point of the trial, was assessed both by foot score (all four feet) and foot volume (volume displacement of both hind feet). The positive control meloxicam effectively ameliorated joint swelling as determined by reduction in foot score (Figure 7a), which is a measure of the clinical severity of the inflammation with zero being the normal state. PC700 and to a lesser extent PC500 produced significant anti-inflammatory effects (Figure 7a) (82.2%control ± 0.89 (mean ± SEM) and 86.8%control ± 1.27, respectively).

With regard to the foot volume change (Figure 7b), which is a measure of the extent of swelling of the foot, the positive control meloxicam was very effective in ameliorating joint swelling. PC700 showed a significant, although milder, anti-inflammatory effect while there was no significant effect with PC500 (85.1% control ± 0.93 (mean ± SEM) and 91.9% control ± 1.78, respectively). Neither PC600 nor G600 were effective in this model. Although there were some tendency towards a reduction in foot score, results did not reach significance (Trial B; foot scores 90.9% control ± 1.83 (mean ± SEM), and 94.5% control ± 2.10 respectively; foot volumes 95.5% control ± 1.99 and 99.2% control ± 1.78, respectively).

#### 3.2.2. Carrageenan-induced Paw Oedema

There was no significant difference in food intake between the treatment groups over the period of the trial (ANOVA, Tukey HSD post-hoc analysis). Carrageenan injection into the hind feet of rats induced a rapid inflammatory response, as observed by an 80% increase in foot volume in the control group (79.8% ± 0.71 (mean ± SEM), *n* = 16) 4h post-injection. Meloxicam administration two days prior to carrageenan significantly reduced the inflammation as measured by a 31% reduction in foot volume compared to the control (68.7% control ±1.46 (mean ± SEM, *n* = 16, *p* < 0.001)), although the response to meloxicam in this acute model was not as marked as in the longer-induction adjuvant model (63% reduction, Figure 7b). There was a 10% reduction in the foot volume of rats fed the G600-supplemented diet, compared to the control; however, the result did not quite reach significance (90.5% control ± 1.25 (mean ± SEM), *n* = 16, *p* = 0.072). The PC600 and PC700 supplemented diets had no effect on foot volume changes induced by carrageenan (data not shown).

## 4. Discussion

Ascribing an anti-inflammatory effect to any one component or class of component within a complex food substrate is not straightforward, as there may be bioactive synergies or even antagonism at play within the milieu. However, by using selectively enriched fractions it is at least possible to determine which group, or groups of components may contribute to an effect, and further, whether they might have potential as targeted nutritional interventions to modulate inappropriate inflammatory responses. The inflammatory response is multi-layered and involves recruitment of an array of inflammatory cytokines and other mediators in response to infection or injury. However, if these factors are not down-regulated appropriately following resolution of the insult or injury, or if the response is sustained, the inflammation can be become chronic and damaging [25]. In respect of the five inflammatory markers described in this report, IL-1β, a key inflammatory cytokine [45], is implicated in osteoarthritis (OA) [46], and also plays a pivotal role in the pathogenesis of numerous other acute and chronic inflammatory diseases including atherosclerosis, type 2 diabetes, and neurodegenerative disease [67]. The serine proteinase NE, secreted by neutrophils and macrophages during inflammation, has been implicated in lung disease and progression [44,68], while the inducible inflammatory mediator COX-2, a primary target for non-steroidal anti-inflammatory drugs (NSAIDs), has been implicated in arthritis, atherosclerosis, cancer and the neuronal cell injury involved in Alzheimer’s disease [53,69]. In OA, excessive amounts of NO produced largely by inducible nitric oxide synthase (*i*NOS) inhibit matrix synthesis and promote its degradation, while reaction of NO with reactive oxygen species such as SO promotes cellular injury and cytokine-induced apoptosis [70]. Similarly, SO produced at high levels during the respiratory burst phase of the inflammatory response, can be toxic to tissues and result in endothelial damage [41].

The hierarchy of the immune response is such that the expression of one pro-inflammatory mediator is generally regulated through another [45]. However, any of the individual mediators in an inflammatory pathway may act as targets for therapeutic intervention. In this paper, we have presented a collation of data on the effects of various MFGM preparations on the in vitro and in vivo activity of the above key inflammatory mediators with the aim of comparing the patterns of response and gaining further insights into the potential of these fractions as nutritional anti-inflammatory interventions.

From the available data a number of the MFGFM fractions demonstrated a broad range of anti-inflammatory activity rather than being selective towards expression or activity of one particular inflammatory mediator, suggesting the potential for use in a wide range of anti-inflammatory indications. However, level of inhibition was generally more marked against IL-1β. IL-1β is implicated in inflammatory processes at multiple levels [45], and is a pivotal cytokine, affecting the expression of other pro-inflammatory mediators such as *i*NOS, COX-2 and reactive oxygen species (ROS) (e.g., SO) in diseases such as OA [71]. Although it is possible that IL-1β expression might have been a primary target of inhibition in some fractions, the patterns of inhibition were, however, complex and in the case of NO, inhibition was more marked at lower doses with an inverse dose-response observed for all MFGM substrates tested. In the case of the phospholipid fractions PC600 and PC700, there was in fact a significant stimulation of NO production at the higher doses. Given that some of the MFGM fractions have quite varied compositions, the reason for this consistent pattern of activity is not clear. It is possible, however, that all preparations have various other factors in common which when presented at a threshold dose counteract and override inhibitory activity. In the assays described herein, monocytes and neutrophils were activated with LPS and PMA respectively, to stimulate upregulation of pro-inflammatory markers. However, MFGM fractions were pre-incubated with the cells (or whole blood) prior to addition of the inflammatory agent, so it is possible that the inflammatory response may have been modified at the level of cell activation or enzyme expression, or as in the case of NE, release from neutrophil granulocytes. The results from the assay of isolated enzyme human neutrophil elastase, although limited, provide an insight. The parent MFGM preparation, BSP which contains both protein and lipid components, was effective at inhibiting NE in the activated neutrophil assay, while the protein-enriched fraction BPC70 was strongly and dose-dependently inhibitory of HNE in the isolated enzyme assay. The ganglioside-enriched phospholipid fractions, G500 and G600, were ineffective in the latter assay at comparatively high doses, and yet both were inhibitory of NE in the neutrophil assay, as were MFGM phospholipid fractions in general This suggests that there may have been dual mechanisms of inhibition at play, with the protein component of MFGM directly affecting enzyme activity, and the lipid component acting at an earlier step in the pathway of NE release from neutrophils. While it is difficult to draw conclusions in the absence of a full spectrum of data, it appeared that of the MFGM fractions studied, the protein fraction BPC70 had the least effect on inflammatory mediators expressed in activated monocytes and neutrophils.

Comparing the profiles of activity between different MFGM preparations, some overall trends can be observed. None of the phospholipid or ganglioside-enriched fractions were inhibitory towards the constitutive enzyme COX-1 while being inhibitory, or tending to be inhibitory, of COX-2. The parent MFGM preparation BSP, containing both protein and lipid constituents, tended to be inhibitory of COX-2 across the dose range, although inhibitory of COX-1 only at the lower doses tested. At the single dose tested, the BPC70 fraction, containing predominantly MFGM membrane protein with lesser amounts of phospholipid and ganglioside, had no significant effect on either COX-1 or COX-2. This suggests that the COX-2 inhibitory activity of MFGM resides largely in the phospholipid fraction, and furthermore the null effect of these fractions on COX-1 suggests that there is either a relative selectivity towards the COX-2 isoform, or COX-2 enzyme activity is attenuated via another mechanism. 

Within the phospholipid-enriched MFGM groups PC500, PC600 and PC700, the increasing order of phospholipid enrichment is PC500 < PC700 < PC600. While PC500 and PC700 demonstrated broadly similar inhibitory activities across the range of inflammatory mediators tested, levels of inhibition seen with PC600, for those mediators assayed in common, were substantially higher. This supports the notion that the major anti-inflammatory activity lies largely within the phospholipid portion of the MFGM. This is perhaps not surprising since phospholipids administered in the diet have been reported to have anti-inflammatory effects [1,8,25,30,72] and phosphatidylserine (PS), a constituent of the MFGM phospholipid fraction, has been shown to inhibit the production of inflammatory mediators IL-6, IL-8 and PGE2 in vitro, as well as alleviate carrageenan-induced inflammation in rats when co-administered with the irritant [73].

In general, the ganglioside-enriched MFGM fractions G500 and G600 demonstrated similar patterns of inhibitory behaviour towards inflammatory markers, and the levels of inhibition were like those observed with PC600. Although both G500 and G600 products contain some phospholipid, and in fact a higher content of PS than the other phospholipid fractions (Table 1), the overall phospholipid content including SM is significantly less than that for PC600, indicating that the gangliosides themselves may contribute to anti-inflammatory activity. This is in line with observations that dietary gangliosides can interrupt pro-inflammatory signalling via IL-1β in the intestinal mucosa, specifically suppressing production of IL-1β, NO, PGE2, hydrogen peroxide, IL-6 and IL-8, and subsequently alleviating symptoms of intestinal disease [18,74]. Interestingly, in the assays reported here, G500 was highly inhibitory of IL-1β whereas G600 had no effect on this marker. Given that G600 and G600 both inhibited production of NO, SO and NE to a similar degree, this seems an anomaly. However, G600 has a higher content of GD3 (the predominant bovine milk ganglioside, [75] while G500 has a higher content of GM3 (the predominant human milk ganglioside, produced in bovine MFGM fractions by desialylation of GD3) [24]. This suggests that the mono-sialylated ganglioside (GM3), but not the di-sialylated ganglioside (GD3) might somehow downregulate IL-1β expression. A final point worth mentioning in respect of the in vitro data is that the parent MFGM fraction BSP had no effect on neutrophil SO production, while the protein extract BPC70 was inhibitory although not dose-responsively. This supports the notion that there may be anti-inflammatory factors within the protein as well as the lipid components of the MFGM. It has been reported that MFG-E8 (milk fat globule-epidermal growth factor 8), also known as PA6/7 and a major protein component of the MFGM [16], attenuated intestinal inflammation in murine experimental colitis by significantly down-regulating LPS-induced proinflammatory cytokines [76].

Adjuvant-induced inflammation is a well-established and robust model for chronic inflammatory disease and is an important tool for screening and testing of new therapeutics for rheumatoid arthritis. Moreover, it has a proven track record for predictability of efficacy in humans [64,65]. Equally, the carrageenan-induced paw oedema is a useful and reproducible model for testing the efficacy of potential anti-inflammatory agents in acute inflammation [54,60,61]. Both models were used to screen MFGM fractions, selected based on results from primary in vitro screens for their potential as nutritional interventions in the amelioration of inflammatory conditions. Of the four MFGM complex lipid fractions screened as dietary supplements through the adjuvant model (PC500, PC700, PC600, G600) the two phospholipid fractions, P500 and PC700, were effective in ameliorating the delayed-onset foot and ankle swelling which is characteristic of this model. In contrast, of the three complex lipid fractions screened as dietary supplements through the carrageenan model of acute paw oedema (PC500, PC600, G600), only G600 showed a tendency towards anti-inflammatory activity. It is of interest to note here that in a proof of concept clinical trial, G600 as a dietary supplement in combination with a dairy macropeptide, was able to reduce the number of gout flares in gout patients with a history of chronic flares [20]. This suggests that the G600 may well have utility in mitigating acute joint inflammation.

The in vivo behaviours of MFGM fractions observed in this study did not precisely follow the patterns of anti-inflammatory shown in vitro, i.e., in vitro, PC600 was generally more effective against all pro-inflammatory mediators than PC500 or PC700, and ganglioside fractions G500 and G600 were as effective as all phospholipid fractions. However, in vitro activity is not necessarily predictive of in vivo efficacy and the outcome may be dose-dependent. Moreover, since the two rat models are inherently different in terms of the temporal recruitment of inflammatory mediators, the pro-inflammatory targets for the MFGM fractions or components thereof, may differ between models or be influenced differently. It is likely that the anti-inflammatory activity of the dietary MFGM fractions, as observed in the adjuvant model of arthritis (PC500, PC700) and to some extent in the carrageenan-induced paw oedema model (G600), is mediated through the gut mucosa and thence via the systemic immune system. In this context, it was recently reported that dietary SM (0.1% *w*/*w*) attenuated hepatic steatosis and adipose tissue inflammation, and strongly attenuated serum inflammatory cytokines and chemokines in diet-induced obese mice [27] while dietary input of the phospholipids PC and PE, in conjunction with N-acyl PEs reduced the acute inflammatory response in a mice model of carrageenan-induced pleurisy [77]. In addition, it has been reported that dietary supplementation with MFGM protected against intestinal permeability in LPS-challenged mice by controlling the inflammatory response, causing the gut barrier to remain less permeable [78] and similar results had been observed in vitro [79]. In the MFGM supplemented animals, significant decreases in serum levels of the proinflammatory cytokines were also observed. Thus, immune regulation at the level of the gut provides a plausible mechanism through which anti-inflammatory dietary supplements may exert their effects remotely.

## 5. Conclusions

The novel in vitro data and in vivo results presented in the paper confirm and extend observations on the anti-inflammatory effects of MFGM and its derivative membrane protein, phospholipid and ganglioside enriched fractions, and further demonstrate the potential of MFGM as a nutritional intervention to mitigate chronic and acute inflammatory conditions, especially those relevant to joint disease. Additionally, due to the ability of MFGM to influence the activity and recruitment of inflammatory mediators, as observed from our in vitro data and from in vivo data elsewhere, MFGM supplementation may well have application as a moderator of heightened immune responses. In this study, no single class of components appeared to be responsible for all the observed anti-inflammatory effects, which suggests that there is synergy between the various components involved in the MFGM response. Though statistically significant anti-inflammatory activities have been observed in these primary in vitro and in vivo activity screens, clinical impact of MFGM fractions on inflammatory indications will require further study in proof of concept human trials.

## Figures and Tables

**Table 1:** Composition of Milk Fat Globule Membrane Complex Lipid Fractions (g/100 g).

	BSP	PC600	PC700	PC500	G500	G600	BPC70
Total Lipids	17.3	86	84	89	33	30	7.1
Total Phospholipids	7.3	80	60	32.9	17.6	13.3	4.7
PI	0.6	2.6	2	1	2.8	2.5	0.3
PS	0.9	2.4	2.4	1.2	3.6	3.6	0.4
PC	2.0	26.5	19.1	10.6	3.1	1.8	1.5
PE	2.2	21.8	17	10.3	4.9	3.9	0.9
SM	1.5	24.9	16.6	9.2	2.8	1.5	1.5
Total Ganglioside	0.36	-	-	-	1.1	1.7	0.28
Protein	28.5				5.5	6.0	70.8
Lactose	46	6	6.2	4.1	56	58	10.5
Ash	6.4	11	7.4	4.5	5.0	8.3	5.3
Moisture	1.7	1.3	2.0	4.3	3.2	3.5	4.3
Neutral Lipid	10.0	6	24	56.1	14.3	15.0	2.1
GA/(GA+PL)	0.05	0	0	0	0.06	0.13	0.04

Key: BSP, beta serum powder; BPC70, beta serum protein concentrate; PC500/600/700, phospholipid concentrates; G500/600, ganglioside concentrates; PI, phosphatidylinositol; PS, phosphatidylserine; PC, phosphatidylcholine; PE, phosphatidylethanolamine; SM, sphingomyelin. BSP is the parent from which all fractions are derived. Other fractions represent increasing enrichment of phospholipids (PL), or gangliosides (GA) or protein.
